# Genesis of People’s Medicine Centre (PMC) for popularisation of generic medicine: A critical qualitative inquiry

**DOI:** 10.1016/j.rcsop.2024.100455

**Published:** 2024-05-22

**Authors:** Ranjit Kumar Dehury, Imteyaz Ahmad, Manas Ranjan Behera, Varalakshmi Manchana, Parthsarathi Dehury, Deepanjali Behera, Nafisa Vaz e Desouza

**Affiliations:** a School of Management Studies, University of Hyderabad, Telangana 500 046, India; b School of Public Health, Kalinga Institute of Industrial Technology (KIIT) Deemed to be University, Bhubaneswar 751024, India; c School of Medical Sciences, University of Hyderabad, Telangana 500 046, India; d School of Public Health, Asian Institute of Public Health University, Bhubaneswar, India; e Goa Institute of Management Panaji, Goa, India

**Keywords:** PMBJP, Janaushadhi Kendra, Low-cost medicines, Entrepreneurship, Health policy, Universal health coverage

## Abstract

**Introduction:**

The concerns of inaccessibility to essential medicines in India are well-studied and documented. Pradhan Mantri Bhartiya Janaushadhi Priyojana (PMBJP) is one of the policy initiatives to address the inaccessibility of essential medicine. Janaushadhi Kendra (People's Medicine Centre), which is part of PMBJP is being enquired in a limited way to understand its effectiveness. The province of Odisha has been chosen as the study area for the evaluation of People's Medicine Centres.

**Objective:**

The present study intends to inquire into the nature of People's Medicine Centre ownership, pharmacists' motivations and incentives to engage in business, perceived customers' trust and satisfaction, scheme beneficiaries, and challenges.

**Methods:**

A qualitative research approach has been adopted to evaluate the broader subjective accounts of the pharmacists and People's Medicine Centre. An open-ended interview guide was used. The topics of ownership, motivation, incentives, trust, satisfaction, perceived benefits, and challenges has been recorded from the participants. A total of seventeen in-depth interviews were conducted in the province of Odisha, India.

**Results:**

The study found that the ownership of People's Medicine Centre was of two types in the province of Odisha: public-NGO-owned People's Medicine Centres and public-private-owned People's Medicine Centres. The financial incentive provisions in the scheme attracted the private pharmacists. Pharmacists highlighted about the lower price of generic medicines compared to branded medicines, which is very popular among patients. They also pointed out that there is no difference in the efficacy of both medicines. The attitude of physicians, especially private physicians, were considered problematic for popularity and acceptance.

**Conclusion:**

The People's Medicine Centres in Odisha established themselves as trusted outlets despite physicians' unfavourable attitudes. Although the centres have not reached the required geographical coverage, economically developed regions have large number of centres, while backward regions have minimal presence. The scheme needs to be more conducive to the welfare of the masses living in remote and rural areas.

## Highlights

• Regional disparities in People's Medicine Centre (PMC) are found in Odisha.
• The PMC ownership model has undergone an ownership transformation post-2015 and resulted in the large-scale operation.
• Pharmacists perceive PMC and branded medicines are same in quality but hugely differ in price.
• Effective advertisements by PMC and trust building among customers are long processes for PMC entrepreneurs.
• Private physicians are resisting the PMC medicines, but PMC pharmacists are convincing the customers to use.

## 1 Introduction

Medicines are life savers, and their prices are a big concern across the globe. In global south and emerging nations where most people lack financial means, the high costs of vital medicines significantly lower the quality of life.^1^ Due to expensive medications, Indian households incur a significant share of out-of-pocket (OOP) costs.^2^ An estimate shows that about 70% of the Indian population spends 10–20% of their total income on health expenses, which is burdensome financially.^3^ Maintaining treatment compliance with psychotropic drugs helps patients recover from mental illness. One of the causes of low compliance to psychiatric medication is the high cost of the drugs, which many people from lower socioeconomic classes cannot afford, particularly when they are needed for an extended period.^4^ Therefore, it is crucial to dispense high-quality medicines in sufficient quantities and at an affordable price to encourage treatment adherence.^5^ A study on private pharmacies as healthcare providers in Odisha find that a large population prefer to use private pharmacies as compared to other public health providers, thus increasing their Out-of-Pocket (OOP) expenses.^6^

PMBJP scheme, which ensures access to quality medicine, is the policy initiative to the inaccessibility of essential medicines in India.^7^ Branded medicines are often sold at much higher prices than their unbranded generic equivalents, even though they have the same therapeutic value. In a country like India, where poverty is widespread, this is a major problem for accessibility of medications. To address this problem, the Pharma Advisory Forum in 2008 decided to launch the Jan Aushadhi Campaign (medication for common citizen). The campaign aims to make quality generic medicines available at reasonable prices through dedicated sales outlets called Pradhan Mantri Bhartiya Janaushadhi Kendra (PMBJK) or ‘Janaushadhi Kendra’ in various districts of the country, which has been abbreivated as People's Medicine Centre (PMC). The campaign started in 2008 itself.^8^

In essence, this is vital for the growth of healthcare in a manner that addresses the population's requirements while guaranteeing accessibility, affordability, and the quality of services, all of which are pivotal in advancing the country towards achieving universal health coverage (UHC). The PMBJP scheme is a broad subject of study in this regard. The present paper focuses on one aspect of the PMBJP scheme called PMBJK or ‘Janaushadhi Kendra.’ The terminology ‘Janaushadhi Kendra’ is a set of two Hindi words, the literary translation of which is ‘People's Medicine Centre.’ Hence, it is abbreviated as PMC.

PMCs are medicine outlets across India started under the aegis of the PMBJP scheme by the Ministry of Chemicals and Fertilizers, Government of India. These PMCs are the outlets that make the scheme functional on the ground. In the beginning (in 2008), the scheme was started in selected districts of India. Later, it was planned to expand one PMC in each of 630 districts of India with further expansion to district sub-divisions, as well as major town and village centres by 2012.^9^^,^^10^ The scheme was initiated by the Congress party-led United Progressive Alliance (UPA) in 2008 and taken forward by the Bhartiya Janata Party (BJP) led National Democratic Alliance (NDA) in 2014. From 2008 to 2014, the scheme did not perform; less than a hundred PMCs were started. The NDA government revamped the scheme, resulting in a sudden jump in the total number of PMCs (more than 9000 till 2022) across the country post-2015.^10^

A book published by the University of Hyderabad^11^ informs about the medicine supply chain for PMCs. The Pharma and Medical Bureau of India (PMBI), the erstwhile Bureau of Pharma Sector Undertakings (BPSU) of India, is the public entity and nodal agency that runs the PMCs. PMBI sells generic medicines to PMCs, maintains the supply chain, and ensures that the PMCs are properly run. The Central Pharma Public Sector Undertakings (CPSUs) is the national-level agency that ensures the functioning of the country's supply chain of quality medicines.

The PMCs have been established throughout India with the plan of opening shops at all public hospitals and reachable locations.^12^ In terms of ownership, the PMCs are of two types: public-NGO owned and public-private (individual) owned. The public role in the PMC ownership is to provide initial funding for infrastructure establishment and maintenance of the supply chain. The role of Non-Governmental Organizations (NGOs) and private partners is to run the outlet on a self-sustaining model. This means that the government will only provide initial financial support, while later, the NGO and private partners will generate profit and run the outlet.^8^^,^^9^ The programme offers store owners incentives with assistance ranging from INR 200,000 to INR 500,000 (2412 to 6032 USD approximately). This is a one-time transfer. The PMBJP scheme provides a 20% discount on MRP (Excluding taxes) to collaborating entrepreneurs in medicine purchases, which is sufficient for the profit margins.^8^^,^^13^^,^^14^ Additionally, there are special incentives for women entrepreneurs, persons with a physical disability (PwD), Scheduled Castes (SC), Scheduled Tribe (ST), ex-servicemen, and any entrepreneurs who open PMC in the identified backward districts, also called aspirational districts in Himalayan, Island territories and India's North-Eastern provinces. These entrepreneurs receive an amount of INR 200,000 (2412 USD approximately) in addition to the normal incentive.^8^

In the healthcare ecosystem, PMCs rely on government support and customers' trust. The objective of the PMC is to provide low-cost quality medicines to all, with emphasis on underprivileged and impoverished sections of the population. In the pursuit of large-scale coverage, PMCs aim to break the widespread misconception that high prices are equal to quality. The means used for the purpose are education and publicity measures. The creation of employment opportunities for unemployed pharmacists is the third objective of the scheme though PMCs.^15^

### 1.1 Theoretical implications

Regional inequality in India is supposed to be reduced by active policy intervention.^16^^,^^17^ Public schemes are such tools of the policy framework that serve the objective of reducing regional inequalities. There is, for instance, evidence of regional inequality in access to immunization in India, which was addressed by government intervention.^18^^,^^19^ PMBJP scheme was formulated to reduce patients' financial difficulties. It is implemented across the country by engaging the non-governmental and private stakeholders. Non-governmental organizations (NGOs) are not-for-profit institutions that help in creating public goods. NGOs are registered civil society organizations, such as the Red Cross Society. Qualified pharmacists who are citizens of India can enter into a contract with the government to establish PMCs. The scheme relied on the private stakeholders to take it to the remote and unserved regions because public and non-governmental stakeholders have logistical and infrastructural limitations. Karahasan et al.^20^ argued that market potential explains regional inequalities in developing countries. The market potential is strikingly visible when public sector distance itself from market-oriented profit-making activities and promote private sector. However, unregulated market due to faulty framework fails the welfare agenda of the state; such as, injudicious distribution of PMCs in a geographical region serves those who are already better served than those who are completely unserved. There are various factors of health inequalities in India, such as education, caste, gender, income, etc.

Regional disparity in accessing health has remained a consistent and homogeneous factor in India and across the globe.^21^^,^^22^ In terms of health outcomes, such as infant mortality rate and maternal mortality rate, regional disparities across Indian provinces are visible.^23^ Distance, transportation cost, and land rent are crucial factors that determine the profit and, hence, the choice of location decision of an entrepreneur.^24^ Head and Mayer^25^ added that the interaction between trade costs and the firm-level scale of economies affects the formation of business agglomeration. Karahasan et al.^20^ use the terminologies ‘centripetal and centrifugal forces.’ The centripetal forces result in firms' agglomeration, while centrifugal forces make firms and consumers disperse. The equilibrium of both forces determines the actual location of firms in the real world.

Apart from the theory of regional disparities in the expansion of PMCs, hegemony theory also has implications for the present study. For a system to run properly, there needs to be a powerful state that dominates the subjects' thought processes.^26^^,^^27^ Similar is the case of physician-patient relations, where the physician controls the patient's behaviour, including medication. People are not ruled by force alone but also by ideas. The ruling ideas happen to be ideas of the ruling class, said Bates.^28^ In the healthcare sector, the hegemony is of physicians and their worldview is consented to (by patients), diffused and popularised by the masses seeking their expertise to get rid of health problems. The physician hegemony is in contradiction with the popularisation of PMC. Brand manufacturers apply a number of marketing strategies to scale-up their product's sales. In the process, they give money to physicians' associations.^29^ Such financial favours, along with massive advertisements, influence physicians' decision-making regarding drug prescriptions.^30^ Hence, a public welfare scheme that provides low-cost quality medicine to patients without favoring physicians' interests fails in its objective of reaching the masses.

The study underpinned these two theories for data analysis and interpretation.

### 1.2 Objectives

The paper aims to study the PMC's presence in the purposively selected districts of Odisha, the nature of PMC ownership, and private pharmacists' motivations and incentives to engage in the PMC business. It is intended to explore the pharmacists' opinions about customers' trust and satisfaction with PMC's medicines. Additionally, the paper aims to explore the pharmacists' perceptions regarding the scheme's beneficiaries and the challenges of running a PMC.

## 2 Materials and methods

The topic of PMC is new and not researched enough. Therefore, the present paper offers an exploratory approach, although it relies on two existing theories that have implications for the study; regional disparity and hegemony theory. There are three main stakeholders in the PMBJP scheme: first, the government agency PMBI; second, the entrepreneurs/pharmacists (NGOs & individuals); and third, the medicine consumers. The present study chooses to explore the motivations and incentives of entrepreneurs/pharmacists. The semi-structured conversations between the researchers and participants tried to capture and highlight different aspects of PMC business. The current qualitative research findings can be helpful in the formulation of a survey questionnaire for future research on PMCs.

Seventeen in-depth interviews with PMC owners and pharmacists were conducted. A list of PMCs was obtained from the PMBJP website maintained by the government of India (janaushadhi.gov.in). The study is focused on the province of Odisha. The province is located on the eastern side of India. Odisha has seen severe incidences of poverty and lack of health care services. A range of factors contribute to poverty in Odisha, including poor education, caste discrimination, low monthly per capita consumer expenditure, food insecurity, poor shelter, and absence of healthcare.^31^^,^^32^ In that case, the presence and functioning of PMCs in the province become a relevant issue in studying access to medicine from the supply side (or pharmacists') perspective. The PMBJP scheme is essential for all the provinces of India. Some of the provinces, including Odisha, are in dire need of such schemes.

### 2.1 Locale of the study

Odisha is an Indian province situated on the eastern coast. It has 30 administrative geographical units called districts.^33^ Data was collected from five purposively chosen districts due to the ease of logistical arrangements and time constraints for the research team. These five districts are in rural, urban, and metropolitan regions. Kalahandi and Rayagada are tribal and rural populated districts and have been known for primary sector activity and extreme poverty for a long time. These two districts are situated in the south of the province. Angul and Keonjhar are situated in the northern part of the Odisha. These two are known for industrial mining and mostly urban settlements. Khordha is an economically well-performing district and the capital region of the province. This district is located on the eastern side of Odisha and is also a coastal district. In this district, tertiary sector activities are performed.^34^ The essence of selecting five districts in three different regions is that PMC pharmacists' motivations, beliefs, challenges, opportunities and presence in different regions could be explored extensively.

**Fig. 1:**
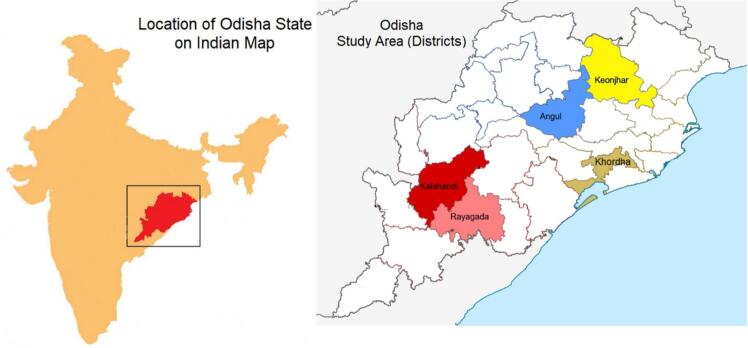
Location of districts selected on the map of Odisha. Note: Map not to scale, only for pictorial representation.

Fig. 1 Provides the location of districts selected on the map of Odisha as study areas. Table 1 shows the number of listed PMCs in selected districts and in-depth interviews in respective districts. There is injudicious and unequal distribution of PMCs in Odisha. Economically well-off districts (Khordha) have the highest number of PMCs compared to deprived districts (Kalahandi and Rayagada). The number of interviews conducted varies according to the relative scope of finding a PMC in the selected districts.

(Source: Authors own representation,)

**Table 1:** Number of listed PMCs in selected districts and in-depth interviews in respective districts.

Sr. no.	Districts	PMCs listed on website⁎ (till January, 01, 2023)	In-depth interviews conducted with pharmacists		
			Owner^1^	Hired^2^	Total
1	Angul	8	0	3	3
2	Keonjhar	10	2	2	4
3	Khordha	38	5	3	8
4	Kalahandi	1	0	0	0
5	Rayagada	2	2	0	2
	Total	59	9	8	17

Source: Authors' representation.

⁎ www.janaushadhi.gov.in

1 Only private individual entrepreneurs.

2 Including PMCs run by Red Cross NGO.

### 2.2 Data sources and ethical considerations

The data about PMCs listed on the PMBJP website has already revealed that the distribution of PMCs is unequal (Table 1). The underprivileged districts of Kalahandi and Rayagada had 3 PMCs listed, while the most privileged district of Khordha had the highest (total of 38) listed PMCs. It led to the application of regional disparity theory in this research. The research sought to explain regional disparity through the source of information regarding the PMBJP scheme, motivation, challenges and opportunities to run a PMC.

The PMBJP scheme aims to challenge the notion that quality is always associated with high prices. The study found implications of hegemony theory by underpinning the phenomenon in the scheme objective, where physicians prefer to market pharmaceutical brands and dictate consumer behaviour.

The interviewed persons were owners or employed pharmacists of the PMCs who had at least five years of experience in the pharmacy. Participants were selected using convenient sampling. Due to rush hour, some potential participants refused to participate in the study. Many others asked to visit some other time. Researchers visited the PMCs and interviewed the pharmacists with their comfort and convenience.

Government documents, pharmacy outlets, newspaper articles, policy briefs, previously commissioned studies and authentic databases were examined to prepare the Interview guide. The interview guide was used as a data collection tool. The researchers put prompt questions/topics and let the participants speak. Many nuances related to PMC medicines came out freely. The data collection approach was mostly deductive. The enquiry about the source of information that led to the knowledge of the scheme was supposed to reveal the information channel, which was active in well-served districts and inactive in unserved districts. The dominance of physicians in dictating ‘quality is equal to high price’ was investigated with the PMC pharmacists' experience of receiving private healthcare practitioners' prescriptions. Some examples of the questions asked are: How do you learn about the PMC scheme? Who owns this PMC centre? Why this PMC is started? What is the thought or motivation behind opening this PMC? Can you tell us what the difference is between the market brand and PMC medicine? Are PMC medicines as efficacious as market brands? What are the physicians' views about the PMC medicines? Who is benefitting most from this (PMBJP) scheme? How much is your margin in the product's Maximum Retail Price (MRP)? Is there any risk or loss in this PMC business? If yes, then what is it, and how is it managed? After conducting seventeen interviews, the researchers found the saturation point reached.

The interviews took place at the PMC centres. The interviews were conducted in the months of January and February 2023. Interviews were voice recorded. The language of the interviews was Odia and Hindi. Two interviewers conducted the interviews. Both the interviewers were public health researchers. They were familiar with the study area. One interviewer was fluent in Odia, and the other was fluent in Hindi. Interviewees were given the choice to speak in either of the languages. During interviews, few interruptions used to occur; for example, customers were coming frequently, and pharmacists had to leave the interview in between for some minutes. Despite breaking the interview flow and paucity of time for a long, in-depth interview, the researchers stuck to the interview guide while keeping the scope of the probe. Further, most of the interviews were carried out post-lunch because, at this time, pharmacists used to have some leisure.

Further, the study followed the COREQ - 32 items checklist to abide by the qualitative research guidelines, which have been provided in the suplimentary section.^35^ Informed consent was obtained at the start of the interviews. Ethical approvals have been taken from the university ethics committee before conducting the study with reference number UH/IEC/2021/158, Dated. 26.08.2021.

### 2.3 Data analysis

Interviews were transcribed in the original languages, Odia and Hindi. A free version of InqScribe was used to transcribe the recorded interviews into text form. Then translated to English. All the personal information of the interviewees was removed from the transcription to maintain anonymity and confidentiality. In the translation, no redactions were made except the actual names of the participants and their identities. The participants were given pseudonyms for data analysis. Language experts were consulted to check and verify the accuracy of the translations. Existing theories (regional disparity and hegemony theory) were the basis for locating the research stand. Topics included in the interview guide were pre-determined themes that were going to test the theories. Codes were created using the transcribed interviews. Sentences and parts of interviews were highlighted with the relevant codes. Table 2 is a detailed description of all the codes and themes created for the study.

**Table 2:** Codes and themes.

Sr. no.	Codes	Themes
1	About PMC	About PMC in Odisha
2	Motivation or inspiration	The Motivation and Incentive to Start a PMC
3	Market competition	
4	Knowledge about PMC	The Difference of PMC and Branded Medicines
5	Trust building	Presence of PMCs, Advertisements and Customers' Trust Building
6	Self-medication	
7	Satisfaction	Trust and Satisfaction
8	Beneficiary	Beneficiaries of the PMCs
9	Loss or dissatisfaction	Challenges faced by PMCs
10	Supply chain	
11	Suggestions	
12	Role of Drug Inspector	

Source: Authors' representation.

**Fig. 2:**
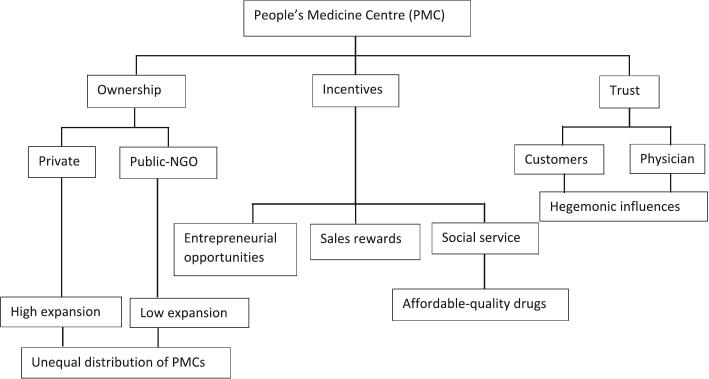
The ecosystem of PMCs in Odisha.

The interview guide served as a basis for the determination of themes. A deductive approach in analysis is utilized to keep the data focused on the research objective. The data was imported into MAXQDA software. It is a product of a Germany-based company called VERBI. The online address of this software is https://www.maxqda.com/. The software helped to organize the data thematically. Twelve codes and seven themes were identified and analyzed, and the researchers reviewed them. The researchers reported the themes and their supporting evidence in a clear and organized manner by using quotes or excerpts from the interviews to illustrate the themes and provide a rich narrative. Fig. 2. Shows the Ecosystem of PMCs in Odisha and its determinants.

(Source: Authors' representation)

## 3 Results

The themes that emerged from the data positioned the study as a new dimensional study in generic medicine, particularly in policy-led generic medicine provision research. The present study focuses on the perspectives of PMC proprietors. It includes the nature of PMC ownership, the motivation and incentive to engage in the PMC business, the perceived differences between market brands and PMC's medicines, the coverage of benefits of the scheme, the emphasis on the presence of PMC, advertisements and trust building among the customers. Customers' satisfaction and the perceived beneficiaries of the scheme revealed the proprietors' worldview about their business. Challenges faced by PMCs are also acknowledged by the proprietors, which shows structural problems in the PMC-subsidized drug model.

### 3.1 About PMC in Odisha

A significant surge in the number of PMCs in Odisha occurred after 2015. Before 2015, there were only a few PMCs, as per the data.^13^ The major difference between pre-2015 and post-2015 PMCs is the nature of ownership. The Central Government franchised PMCs to the District Red Cross (DRC) society across the province of Odisha earlier. Government hospital pharmacists were appointed on deputation to run the PMCs. Ranjan (name changed) was a government pharmacist and was interviewed at a Red Cross-run PMC in Angul. He said,

This scheme is of the central government. During the Congress government, the scheme was launched. In 2010, there was a district Red Cross board in every district's Red Cross branch, also called DRC. Every DRC in different districts was informed to open a counter on behalf of the Red Cross. In 2010, after CDMO's (Chief District Medical Officer) efforts, the Red Cross secretary was appointed as an officer in charge, including one of our physicians…

[Ranjan, Angul, Red Cross PMC]

Ranjan (Angul) further elaborated his position in the PMC as,

So, I am an employee here, and this is a government-owned store; this is not my private store. The Red Cross manages this store, and all of us (the staff in the store) run it. I am working here on deputation. I was there (in the hospital) before, and I was transferred here on deputation. This (store) is under CDMO.

[Ranjan, Angul, Red Cross PMC]

After setting up the system for PMC, the next step was the provision of funds. In the scheme policy, it was decided that the PMC centres would be self-sustaining. However, initial support money was provided to start the business. Ranjan (Angul) said,

They (DRC secretary and public hospital physician) opened a joint (bank) account. Then, INR 200,000 (approximately 2412 USD) was provided (by the government) in that account. After 1-2 years, we again demanded INR 200,000 (approximately 2412 USD) to purchase and invest in the business and in aggregate, INR 400,000 (approximately 4824 USD) was obtained. The sales and profits are updated in the joint account held by our physician and Red Cross secretary.

[Ranjan, Angul, Red Cross PMC]

In the post-2015 era, the rules were changed, and private proprietors were invited and encouraged to get the franchise. Although a surge was recorded in the number of PMCs post-2015, no Red Cross and public hospital collaboration was seen in this period to start the PMCs. Only the existing Red Cross-run PMCs continued. Lilabati (name changed), a female pharmacist in Khordha Red Cross PMC, recalls;

…not a single private PMC was there. (Whatever were there) It could be counted on fingers; like in the Bhubneshwar, there were three: one in Khordha, one in Capital Hospital, and the third one is this. It is the Red Cross office; that is why one is established here while others were started in district headquarters.

[Lilabati, Khordha, Red Cross PMC]

The motivation and information to start a PMC were primarily disseminated through peer networks and advertisements. The monetary support of up to INR 500,000 (approximately 6032 USD) attracted potential entrepreneurs and pharmacists to engage in the PMBJP scheme. Gaining profits in the business is the ultimate goal of any entrepreneur. Therefore, it is not just serving people but engaging in a startup supported by the central government. Existing PMC entrepreneurs pass down a chain of information and motivation to potential entrepreneurs. Dilip (name changed), a PMC owner in Khordha who started his store two years ago, said,

Regarding information, there is a store of my friend; he informed and invited me to start this business, as it is a good business, he said.

[Dilip, Khordha, Private PMC owner]

### 3.2 The motivation and incentive to start a PMC

For individuals, being a PMC pharmacist is an opportunity to earn a positive income. PMC pharmacists provided several instances where operating a PMC was a better entrepreneurial choice to maximize income. Dilip (Khordha) said the scheme gives a 20 % price margin as profit and an additional 2 % to manage losses incurred due to expired medicines. There are provisions in the scheme to motivate pharmacists to scale up their business with increased sales. Dilip substantiated that the scheme includes provisions to incentivize pharmacists to scale up their businesses with increased sales, with PMCs reaching specific sales targets and becoming eligible for 15 % incentives.

Lokesh (name changed) was a PMC proprietor in Keonjhar who had been running his store for over five years and was set to start two more PMCs in Keonjhar at two locations. The growth of the current store incentivized him, and he was willing to start two more stores.

Seeing others and with the support of peers, some started PMCs. Prabeer (name changed) was a pharmacist in Keonjhar, running a generic medicine store called an ‘ethical shop’ for many years. He transformed his old ethical shop into PMC. His elder brother had recently started a PMC in Cuttack, and he helped his brother shift towards PMC.

The PMCs are rapidly flourishing these days, informed Dilip (Khordha). According to him, sales are increasing daily, and it has not been impacted even after opening up another PMC in the vicinity.

Before 2015, there was nothing like motivation and incentives because earlier, it was not a proprietor-based system; the Red Cross was invited to focus on some of the vulnerable regions of Odisha, said Lilabati (Name changed, Khordha). At that time, government hospital pharmacists were appointed on deputation. They were supposed to maintain a joint bank account with the Chief District Medical Officer (CDMO), or the bank account would be operated solely by the district collector. So earlier (before 2015), PMCs were purely government units managed by the Red Cross, and there was no role for any private entity to play. Motivation and incentives can work only in the case of private sector involvement. With motivation and incentive comes efficiency in the services.

Apart from business gains, there was a sense of social service and learning opportunities for some pharmacists to get involved in the JAKs. Gajanan (name changed), a pharmacist in Rayagada, said,

Please see, this is an opportunity to serve the people. Poor get it in very less price, keeping that in view, this store was started. Even before that, we were running only a generic product store. But that was different; this one is a new model. So, by doing that, the people are served here. In Rayagada, people know this is the only shop for quality and low price medication…

[Gajanan, Rayagada, Private PMC pharmacist]

Suraj (name changed) was a young PMC pharmacist in Angul who was quite optimistic about learning new things in the PMC. This is the motivational force behind the shift from branded stores to PMC.

I moved here because I am gaining knowledge about medicines here. For example, when we (pharmacists) see brand names, we remember only brand names and often do not remember the composition. But here, it is different; we see the composition name directly and remember it and its power strength (mg). How much mg is required depends upon the patient's situation, etcetera, we can supervise.

[Suraj, Angul, Private PMC pharmacist]

### 3.3 The difference of PMC and branded medicines

This theme reviews pharmacists' awareness of branded, PMC, and generic medicines. Although these categories have clear differences, it is essential to understand which aspects pharmacists highlight. The price at which PMC medicines are sold differs greatly from the market average of branded medicines. Pharmacists were quick to highlight it. Dilip of Khordha revealed the price difference is as much as 40–50%. Composition-wise, both branded and PMC medicines are the same; there is no difference, said many pharmacists. However, the difference is realized due to a fixed mindset. Arpana (name changed) was a young female PMC pharmacist in Keonjhar. She expressed her belief that;

I think all are the same; the only difference is in people's mindsets. They think this medicine will be best, and if they are caught in negative thinking that this one will not be good, then no matter what medicine we give, they won't accept it. And this is it, what I feel.

[Arpana, Keonjhar, Private PMC pharmacist]

The naming of the medicine differentiates the two; pharmaceutical companies name their products over the original component names, while PMC products only carry the component names. There is no double naming in PMC. Pharmacists appreciated this feature and mentioned this as a major difference. The PMC pharmacist in Angul district hospital, Ranjan, tried to explain the difference with vivid examples;

I will tell you (the difference). Do you know any brands? Do you take any medicine? Do you take Paracetamol? Or Crocin? Or Calpol? Do you know all these? I am telling you because these are well-known. Crocin and Calpol are brand names. And their composition is paracetamol. Isn't it? The real-named medicine is called a generic drug. But that is only its real name. Your name is Gopal. This is your real name; the other pet names are not your real names. For example, in records and on the Aadhaar card (government-issued identity card), the name you mentioned is your real name. Then, that only is called generic medicine. And the brand names are the pet names.

[Ranjan, Angul, Red Cross PMC]

Pharmacists across the PMCs appreciate the relief of memorizing just one name over many. The PMC proposed that medicines must be called by their molecule names rather than creating many brand names. It is a significant step in breaking the brand name hegemony. Knowing molecule names could be empowering to pharmacists. If a molecule is known, it can be easily searched for across various manufacturers, and transparent communication is also possible. PMC pharmacist Suraj of Angul said,

…I am gaining knowledge about medicines here. For example, when we (pharmacists) see brand names, we remember only brand names and often do not remember the composition. But here, it is different; we see the composition name directly and memorize it and its strength (milligram, etc.). How much mg is required depends upon the patient's situation, etcetera, we can supervise.

[Suraj, Angul, Private PMC pharmacist]

### 3.4 Regional disparity in the presence of PMCs, role of advertisements and customers' trust building

The PMCs need widespread awareness among people. In this regard, the most important thing is the presence of PMCs in every nook and corner of the country. Suraj (Angul) emphasized the presence of the PMC for the larger benefit of the people.

I suggest these shops (PMCs) should be increased at a large scale so that People's Medicine Centres could be present in every corner and every village. So that people's dependence on market drugs can be reduced and people become more aware of PMC. For that purpose, there is a need for advertisements in the villages.

[Suraj, Angul, Private PMC pharmacist]

The researchers found a satisfactory number of PMCs in Khordha (38 PMCs) and relatively lesser numbers in Angul (8 PMCs) and Keonjhar (10 PMCs). The researchers also found that Rayagada (2 PMCs) is underserved, and Kalahandi (1 PMC) is an unserved district. Only one PMC was listed on the janaushadhi.gov.in website for the Kalahandi district by the time the study was conducted. That, too, was not functional when visited. The researchers' report mentioned that the only PMC established in Kalahandi, Odisha, was found non-existent.

The research team reached the Kalahandi district of Odisha to interview the PMC owner or the pharmacist at the centre. The address was drawn from the PMC website. Along with the address name of the contact person, the store code and status of the store were also attained. The store was indicated as functional on the online list and located on the campus of the District Headquarters Hospital. The team searched for the PMC but found nowhere. However, some branded medical stores were functional in the same district headquarters hospital campus. On enquiry, the team was informed that there was a government medicine counter a few years ago, but it could not do any business and shut down shortly after, a private medical store said. The team searched for the contact person inside the hospital.

The contact person said it shut down 5–6 years ago. He informed the team that the shutdown happened due to no demand. Because in the same hospital campus, the Niramya scheme of free drug distribution was going on then, nobody wanted to spend on the cheapest even if it cost only five rupees. He also mentioned that losses incurred in the form of excess expiries within two years of operation. Physicians did not prescribe PMC medicines. There was no motivation to run the centre. The contact person was appointed on deputation for two months only. He was a Odisha government employee and pharmacist in the hospital. He also informed the team that the centre was government-owned. He said he could not talk much about the PMC in the hospital compound. He referred the team to a person at the hospital warehouse to enquire more about the centre. The referred person was not found at the warehouse. When the team reached out by phone, he said he could not talk as he was busy in a meeting.

It is important to note that compared to other districts of Odisha, where several PMCs were located, there was only one in Kalahandi, and that too was shut down a long time ago and has yet to be updated on the website. Researchers observed that people were in the queue at the free drug distribution centre of the Niramya Scheme.

No demand for PMC medicine is an outcome of low-awareness at first hand, and the presence of a free drug distribution scheme (Niramaya) on the other hand in this case. Access to free drugs is essential for better health outcomes. However, the poor presence of drug distribution units in a poverty-stricken district is a matter of concern when people stand in queues. A district hospital, along with a free drug distribution centre (Niramaya), can serve only a handful of patients, while a major section of patients in remote areas remain unserved and not able to access the services at the district headquarters anytime they want.

With the need to increase the number of PMCs, awareness is equally important. Awareness is important to inform people to utilize the PMCs and also to make PMCs a profitable venture for private owners. Dilip (Khordha) said,

There should be advertisements. There is not much focus on awareness. They (the government) do, but it is not as much as required. With more advertisements, more people will come. Until now, many people do not know what the PMC is.

[Dilip, Khordha, Private PMC owner]

When a PMC owner says there is a poor advertisement, the government should popularise it. The onus of the advertisement is on the scheme, but the ultimate beneficiaries of the advertisements will be PMC owners because it will attract more customers, and hence, the business will be more profitable. The researchers asked whether the owners were doing some advertisements on their behalf. Carry bags with PMC names printed on them with addresses and flex-boards were a common practice of advertisement by the PMC owners. Dilip (Khordha) mentioned he makes his best efforts on his behalf to popularise his PMC, but he considers his efforts trivial and cannot bring a bigger change. He said;

What we can do! Sometimes, we distribute small souvenirs such as calendars or sometimes visiting cards, that is it. One more thing that the government does (for PMC promotion) is called PMC Day in the first week of March… Health camps are put on for an entire awareness week, and some other activities happen. That is it; nothing much happens… sometimes we distribute (sanitary) napkins in the slum areas... only a few small activities. There is nothing much on a large scale… We have printed a calendar for the new year. One calendar is given to every customer. This is it; what else we can do? There is not much initiative from the government side. After a long time, I saw an advertisement in the newspaper. So this is our approach. Nothing much. That is it.

[Dilip, Khordha, Private PMC owner]

With limited resources, PMC owners were able to advertise on a small scale. PMC Day is a state-declared annual event that PMCs celebrate to promote awareness. The event allows all the PMCs in a region to come together and engage in public activities that bring them to public attention.

Arpana (Name changed, Keonjhar) was innovative in advertising her PMC. She used social media platforms to keep hold of existing customers and attract new ones. She applied the social influence technique to advertise and gain trust among people. She said,

I created a WhatsApp group. Those who come here (at the PMC) and do not find their medicine due to non-availability do not come back next time, even after being informed by fellow customers (that their medicine is available). The advantage of including them in the group is that they get influenced by seeing those who regularly purchase. I keep updating new stocks. Hence, at least two to four new customers are added daily.

[Arpana, Keonjhar, Private PMC pharmacist]

Pharmacists acknowledge that people know well enough about PMCs now. It is not a new thing for them. Trust in PMC has improved as well. The PMC venture is profitable now. Anyone who starts a new centre today would not have to face the challenges like three years ago. A Red Cross-run PMC situated in the compound of a government hospital in Khordha is a well-known and well-functioning centre. Rahul (name changed) was the Red Cross-run PMC (Khordha) pharmacist; he emphasized the lifetime of the Kendra and the trust built in that period,

It is very good... this is not a one- or two-year-old store but a twelve-year-old one. People were not aware of that much earlier, but now everyone knows that inside the Capital Hospital campus, there is a PMC. Whosoever you ask, they will tell you that low-cost medicines are sold here.

[Rahul, Khordha, Red Cross PMC]

### 3.5 Trust and satisfaction

The post-2015 PMCs in the initial years earned almost negligible profits due to a lack of trust. PMC owners and pharmacists went to extremes to survive and exist in the market. Manohar (name changed) was a private PMC pharmacist in Khordha, he recalled;

Customers were not coming for (the first) year; we used to open the shop every day and sit idle, but now people are coming.

[Manohar, Khordha, Private PMC pharmacist]

Suraj (Angul) shared his experience. In his initial days, he went beyond the professional limits of entrepreneurship and tried to sell PMC medicine by differing the payments for later as customers did not trust the PMC's efficacy. He said,

No, they (the customer) did not (trust), and we kept trying to convince them. They demand the same branded medicine. We explain to them and request that they please take one month of medicine for free, try it, and take a test afterwards. If you get the desired results, you may continue with us, so these were the things we did.

[Suraj, Angul, Private PMC pharmacist]

After two years of running the PMC, Dilip (Khordha) said that the majority of the customers do not complain about the drug quality. A good number of regular customers rely on PMCs for chronic diseases medicines, such as diabetes and blood pressure.

The physician's role in prescribing PMC medicines is critical. Unless physicians prescribe and suggest patients consume PMC medicines, it is difficult to see a common social acceptance. Physicians are the driving force behind the demand for medicines. Therefore, physicians are also important stakeholders in the popularisation of PMC medicines. If physicians are sensitized towards patients' economic and financial vulnerabilities and made aware and informed about PMC provisions, the challenge of low awareness will be addressed easily.

Ranjan (Angul) narrated his long battle to make government hospital physicians prescribe PMC medicines. He remembered that, in 2010, when his Red Cross-run PMC was started, the physicians of the government hospital where the PMC was situated outrightly refused to trust the PMC medicines' efficacy.

…the PMC counter when it was started, physicians used to write in brand names. So, we had to make an extra effort to run this store; we studied a lot (to match molecules in branded and PMC medicine) and even had to fight. Physicians opposed PMC; they said these (PMC) medicines were unacceptable. Sell branded medicine; otherwise, we will not accept it. Many times, medicines were returned. Then I met them (physicians) and asked why, sir? Is this (PMC) medicine not good enough? Would you testify that this (PMC) medicine is not good? This is forwarded by central government order. Why do you not approve of it? So, these physicians did not recommend PMC medicine. Then I raised this issue in the review meeting in New Delhi; there was a country-level review meeting on PMBJP.

[Ranjan, Angul, Red Cross PMC]

To deal with the low physicians' confidence in PMC, after the review, the Central Government passed an order instructing the province and district collectors to ensure that physicians only write the molecule names. According to Ranjan (Angul), physicians did not change immediately; a tussle continued for another three to four years, but now, eventually, the situation has changed, and no government physician prescribes brand names.

PMC medicines are prescribed by government hospital physicians only; private practitioners abstain from PMC. Manohar (Khordha) revealed the situation;

The majority of government physicians prescribe generic (molecule names) while others do not prescribe in generic names. We have to check the composition and also convince the customers. People are taking it that way.

[Manohar, Khordha, Private PMC pharmacist]

Lokesh (Keonjhar) brings to the physician's role the differentiation of branded and PMC medicines. According to him, a physician's role is to prescribe medicine and not market any brand. He also tried to explain the phenomenon with an example;

Branded is what… the inside thing… the physician will prescribe you as per MCI, Medical Council of India, the physician will prescribe you half litter milk to be taken at night. The physician will not mention that you have to take Amul, Omfed, Pragati milk (Milk brands) or whatever is locally available. A physician will prescribe half a litre of milk daily before bed. A physician cannot mention that you have to take Amul milk,once a physician prescribes Amul or any other (brand), that is called branding.

[Lokesh, Keonjhar, Private PMC owner]

His point was that only the molecule is important, not the manufacturer or service provider. A physician should prescribe the molecule and not the manufacturer's name. However, people trust physicians and do not practice their right to choose an appropriate manufacturer for them. The problem with consumers' mindset is that things are valued as per their price; the costlier the thing, the more trustworthy or efficacious it is assumed. Due to the extremely low cost of PMC medicines, people think PMC medicines are untrustworthy or are low-efficacy drugs. However, social influences can change the perceptions dramatically, as explained by the Red Cross-run PMC pharmacist Ranjan (Angul);

…those few who are poor show interest, and a few others were saying this is so cheap (they) will purchase from outside (branded medicine). This was a mindset: this (PMC medicine) will not work, so one should purchase from the market, they were saying. Rich people would say, huh, we will not take (medicine) from here (PMC), leave it! So there were people from both sides; a section of the poor, as well as the rich, had perceptions that were not in favor of PMC medicines. However, this disfavor is no longer present because society follows the educated section in the community. Educated people provide direction. Lecturers, advocates, teachers, and whoever is in a good, respectable position started preferring the PMC medicines. This is very good; many other people are influenced after seeing them… So this mindset has changed to an extent, and now they no longer dislike (PMC), there is no problem at all.

[Ranjan, Angul, Red Cross PMC]

### 3.6 Beneficiaries of the PMCs

Once the trust is built in the PMC medicines, the venture becomes a beneficial enterprise for both buyers and sellers. The pharmacists were sure and confident that the PMCs primarily benefitted households of every section, whether rich or poor. Pharmacists assert their benefits also. Divya (name changed) was a young female pharmacist in Rayagada. She was happy that at this young age, she owned a PMC and managed it herself. Instead of being an employee somewhere, the PMBJP scheme has given her an opportunity to run a business unit, which is more empowering than being an employee anywhere.

However, customers' benefits are much greater than the pharmacists', said Dilip (Khordha), Gajanan (Rayagada), and Ramesh (name changed), a PMC owner in Angul. Ranjan (Angul) descriptively explained the level of benefits reaching the patients by directly comparing the market brands with PMC products;

Now customers are coming themselves, they say, give this medicine, this medicine is good, and available at cheap rate. If you compare the two, for example, this Nimesulide tablet is 100mg, and it is called Nice in the brand. Everyone knows Nice. Nice for one tablet costs INR 6 to 7 (0.072 to 0.084 USD), and the minimum is INR 6 (0.072 USD). But how much does it (Nimesulide tablet) cost here? Here, it costs sixty paisa for one tablet. Six rupees for ten tablets; therefore, sixty paise is needed for one tablet. Then it becomes ten times cheaper than the branded one. So this is the benefit of purchasing from here.

[Ranjan, Angul, Red Cross PMC]

The extraordinary situation of the spread of COVID-19 saw an unprecedented decline in income, not just for the poor but also for the rich class. Medicine, as the most essential commodity of that time, was causing extreme financial burdens on households. PMC brings huge relief to customers' financial conditions. Arpana (Keonjhar) described the COVID-19 situation as,

The most benefitted are the poor people because when they buy medicine, a single item costs anything from INR 200 to 500 (approximately 2.41 to 6.03 USD); they do not have much purchasing power, so they are the most benefitted group. Also, for rich people, when their salaries were cut down during the CORONA, only 30% or 40% of salaries were given. So, they also needed some sort of support for home expenditure and all the other things. People can manage with INR 50,000 (approximately 603.19 USD) and INR 5000 (approximately 60.32 USD) also (as monthly income). Therefore, rich people were able to manage their medical expenses within INR 5000 (approximately 60.32 USD) due to PMC. Everyone benefitted from this (scheme).

[Arpana, Keonjhar, Private PMC pharmacist]

PMCs have made medicines so affordable that a household can manage medicine expenses with other daily expenses, said Suraj (Angul). For private pharmacists, chronic disease (such as diabetes, blood pressure, heart diseases, etc.) medicines bring long-term customers. PMC is mutually beneficial for both sellers and customers. Sellers get good business, while chronic disease customers get quality medicine at extremely low prices. An old and well-established PMC is a substitute and not complementary to another state-sponsored free drug distribution scheme called ‘Niramaya’ in Odisha. People's demand is often turned down due to drugs' non-availability at ‘Niramaya’ stores. Getting all the medicine at one stop is convenient and less troublesome for customers. PMCs are becoming such an option that they can provide almost all medicines, said Rahul (Khordha). PMC medicines are the best-fitted response to the market's parallel economy of counterfeited drugs. Along with market brands, counterfeit drugs are also sold in the market; despite strict regulations, the problem has not been solved. As PMC is selling very cheap drugs, there remain no incentives for counterfeit agents, said Lilabati (Khordha). This is an important reason that people have turned to PMCs as one of the best alternatives in Odisha. PMCs have a reputation as government-sponsored stores that sell only quality medicine; therefore, the satisfaction and sense of being benefitted is much higher among PMC customers, Suraj (Angul) discussed.

### 3.7 Challenges faced by PMCs

The pre-2015 PMCs functioned along with the post-2015 PMCs without any problem. Out of seventeen PMC interviews, only three PMCs were found to be established in the pre-2015 era. One among them was in turmoil and asked to justify their presence on the government hospital premises by the district magistrate. Rahul was the pharmacist of the PMC in question, which had been managed by the Red Cross Society since 2010. The district magistrate himself was responsible for managing the PMC's bank account. He was the authority to issue bank cheques for order payments. The magistrate had stopped payments for the last six months; therefore, there was a lack of stock at the PMC and a crisis-like situation. The magistrate was agitated to see the PMC within the hospital premises despite the ‘Niramya’ store on the same hospital campus.

Apart from the one PMC mentioned above, others were not facing such an existential threat. Financial losses due to expired medicines are very common across medical stores, not just PMCs. The mechanism to protect PMCs from expired medicines losses was in place; however, many PMC pharmacists said it was not enough. The unprecedented situation of COVID-19 incurred the losses of thousands of rupees, said Ranjan (Angul),

Losses are happening. How! They are all our staff (pointing towards manpower engaged in the PMC), and they know everything. I am in charge here. Four staff are there [not clear]. So we four people know everything. The recent order (issued by the authority) tells to purchase the stock only that much which a PMC can sell, and not more than that, (because) they (PMC supplier) will not accept the returned (products). The government is also at a loss. Often, shortages happen, and medicines remain unavailable for longer, so I bring some medicines in excess. During the Corona crisis for three years, so many drugs were expired. Patients could not come, and those who were regular also stopped coming. Therefore, many medicines were wasted. That loss I have to bear from my pocket. Who would believe it? No one was ready to listen, neither the collector nor the Red Cross. No one was listening, and we were here to save something; I made (fake) bills. I wrote bills prior to the expiry dates in the name of (fake) patients and disposed of the drugs.

[Ranjan, Angul, Red Cross PMC]

There were not just expiries of drugs; there is no return policy provision, no matter if there is a valid reason to return. Arpana (Keonjhar) narrated the problem,

It is said there is a return policy, but it has not been implemented yet. They (the suppliers) ask for batch numbers and other details. We provided all the details, but there is still no return settlement from their side. If some medicines are ordered twice by mistake, there is no return or order cancellation policy… The profits decline due to this as well.

[Arpana, Keonjhar, Private PMC pharmacist]

Most PMC pharmacists have learned how to minimize their losses due to drug expiries. Rahul (Khordha) was quite confident about his principles to avoid these losses, he said,

It is expected that only those running items that are demanded regularly should be stored. There should not be storage of all the available items; otherwise, expiries are inevitable, and losses will be incurred. Only those products that are sold at least once daily should be made available. So, I follow this principle; therefore, I face negligible medicine expiries in my pharmacy.

[Rahul, Khordha, Red Cross PMC]

PMCs in remote and underdeveloped areas, such as Rayagada, face regular shortages of supplies. Due to a poor supply chain system, stocks have vanished for many days and months. Gajanan (Rayagada) had to rely on market generics to fill the gap. However, the PMBJP scheme has a condition that PMCs cannot sell branded and market generic medicines. In this regard, Gajanan said,

See, according to this rule, I am not going to get this (PMC supply) anyway (whenever required). Therefore, I cannot shut down my shop. I have to bring other products, right? If I bring branded medicine, people will not buy it. Once they are habitual of this (PMC products), they can hardly manage for 10-15 rupees extra, if I bring branded only that cost 100 times more. They (customers) are not going to buy it. I cannot shut down my shop. For that reason, we also keep (market) generic. I get generics in the market easily, but this (PMC medicine) supply is tough to get. There are long delays in supplies.

[Gajanan, Rayagada, Private PMC pharmacist]

## 4 Discussion

The PMCs were introduced in 2008. The fifteen-year-old scheme has witnessed radical changes in its development. The primary and most significant change has been the evolving ownership structure. Prior to 2015, no private PMCs were present; the government and Red Cross Society partnered to establish PMCs across the province of Odisha.^36^ These old PMCs were found within the premises of a few government hospitals. Therefore, their span or penetration within the population was limited. After 2015, the private proprietor-based PMC model was introduced. The change was necessary to serve the objective of the scheme. The number of PMCs jumped from 80 in 2014–15 to 9303 in March 2023 (according to the PMBJP website; janaushadhi.gov.in). The proprietor-based model is extremely successful because it combines the public and private sectors efficiently, where the People's Medicine Centre becomes self-sustaining and does not burden any stakeholders. PMCs expanded in cities and urban centres but were not found in rural and underprivileged areas till the date of data collection. Despite the structural reform in the PMBJP scheme regarding ownership, all the sections of the population were not served. The distribution of PMC outlets in Odisha remained uneven. However, other private pharmacies in Odisha were enjoying better customer trust due to a better stock of essential medicines than public primary-care facilities.^6^ This reveals that there are untapped market opportunities across the province of Odisha for pharmacies, which PMCs can capture.

However, PMCs of the pre-2015 era (public-NGO PMCs) are in a crisis. The data from this study reveals that the lone PMC in Kalahandi district was shut down a while ago due to its inability to compete with a parallel provincial government scheme known as Niramaya (provision of free medicine for health and wellness), which provides free drug distribution. Similarly, another PMC running in the compound of the public hospital in Khordha was troubled due to the presence of Niramaya Center in the hospital. Therefore, there is a need to reconsider the old PMC ownership model. The old PMC model relies heavily on public support; for example, PMC finances are managed by district collectors. District collectors manipulate the role of PMCs in a public healthcare system by prioritizing the other free medicine distribution schemes. Hence, they interrupt the finances of the PMCs under them. There are other smoothly functioning public-NGO PMCs where collectors did not disrupt them. Ideally, when a PMC can self-sustain, it should not be controlled by any external authority like collectors. The new public-private model is completely autonomous and free from such an authoritative intervention.

For any entrepreneurial activity, the lack of capital as an initial investment requirement is a major challenge in India. Bank loans pose many conditions, including repayment of loan instalments on time. Any business activity is not free from risk. Therefore, a direct transfer of INR 200,000 to 500,000 (approximately 2412 to 6032 USD) is a big support under the PMBJP scheme. PMC, as a startup, has positively impacted the income of the private PMC proprietors. Chaudhary et al.^37^ found it true to be its motto, “Seva bhi Rozgar bhi,” which translates as “service as well as employment.” The essence of this motto is that by opening a PMC centre, one not only secures employment for himself but also contributes to nation-building through his service. It is said that PMC customers were greatly relieved by the provision of low-cost quality medicine.

There could be various differences between PMC and branded medicines, such as cost, quality, trust, satisfaction, etc. The PMC pharmacists emphasized the cost difference as the most prominent one that sets their PMC outlets apart from other private medicine stores. It is important to be relevant in the drug market by entrepreneurs, especially in serving customers. Hence, a PMC centre provides that relevance with 50% - 90% cheaper drugs than the open drug market. The PMCs are unique because they challenge the ‘brand-name’ game played by manufacturers. PMC owners hail the government's effort to reduce medicine prices in the form of PMCs; at the same time, they criticize private physicians and service providers for promoting branded generics. However, Roy and Rana^7^ blame poor efforts from the policymaker's side to popularise the scheme among service providers and patients and dissolve the confusion regarding unbranded generic and branded generic names of medicines. Within the branded and unbranded generic debate, efficacy and safety are the big concerns. For service providers, the efficacy and safety of medicines are the differentiating criteria for quality drugs, irrespective of PMC medicines or other market brands.^7^ Therefore, educating physicians about the quality of PMC medicine is a pending task. Quality and trust in the health sector are less negotiable; high price and high quality are considered complementary to each other. Therefore, breaking that belief and perception is a challenge for the PMBJP scheme.

However, the PMBJP scheme has come a long way; ample research and evidence have been generated that establish PMC medicines as safe and efficacious.^37^ The PMC pharmacists in Odisha claimed that only government hospital physicians prescribe molecule/salt names, while private physicians do not prescribe PMC medicines. Thawani et al.^14^ counted physicians' disinterested attitude towards PMC as a major constraint that stops the benefits from reaching the people.

The spread of PMCs is found to be unequal across the province. The number of PMCs has increased rapidly, but the distribution is unequal. The radical changes that were brought in the scheme in 2015 reformulation, such as a change in ownership, were an important step. There was a shift from public-NGO-owned PMCs to public-private-owned PMCs. Private pharmacists were supposed to act according to the centripetal and centrifugal forces mentioned above. However, centripetal appears more powerful than centrifugal force in the case of PMCs. Public-private ownership flourished exponentially in cities but remains dormant in remote and underprivileged districts of Odisha. In Rayagada, only two PMCs were found, and that too was found in the district headquarters. Kalahandi is one of the most backward districts in terms of development parameters, and it has no PMC. The economically performing district of Khordha, is highly served by the PMCs. The PMBJP scheme's objective seems neglected when it is meant to serve the poor first, but it actually serves the rich. Despite the new incentive plan that facilitates women, differently abled persons, Scheduled Caste (SC), and Scheduled Tribe (ST) entrepreneurs, including entrepreneurs establishing PMC in aspirational districts, Himalayan, Island territories, and the north-eastern provinces,^13^ a severe regional disparity is observed in Odisha regarding PMCs. To change this situation, registered private pharmacists in unserved districts like Rayagada and Kalahandi can be identified and informed about the scheme and invited to start PMCs in the selected districts. A grievance redressal system should be put in place to identify the regional challenges and address them on a priority basis.

Srivatsa, Srinivas and Marathe^38^ emphasized screening of patients with poor and wealthy characteristics. They proposed a model that gave an efficient way to target the poor only to avail the benefits of PMCs. They concluded that there is no need to put effort into advertising PMC. By doing so, the rich will continue to consume branded drugs while only the poor will benefit from the generic medicine provided by the government despite having low confidence in the generic. There is a serious shortfall in the first condition of the study, which is that only the poor are to be targeted while screening out the wealthy patients completely from the potential PMC beneficiaries. This approach is positively discriminating against one section of the population (who is wealthy). There is an old debate about the classification of the poor and wealthy classes in India.^39^ It is a complex task to work on. At the same time, this model by Srivatsa Srinivas and Marathe^38^ totally overlooks the underserved and unserved regions in the country where the population is homogeneously poor. Not advertising the scheme will never enable the people to seek the benefits of the scheme.

Patients show an unshakable trust in the brands they consume. They are ready to bear the switching costs for prescription drugs to a long extent. The brand loyalty for patients is so high that manufacturers never intend to lower the prices.^40^ The high-priced brands are not innovators. They, too, are generic manufacturers, but they exploit the consumer attitude that the consumer will continue to have apprehensions against low-cost, unbranded generics.^40^^,^^41^ There are determinants essential to brand loyalty in pharmaceuticals; brand credibility, communication, experience, satisfaction, identity (self-image), and perceived value are some of the factors.^42^ PMCs in Odisha have already faced this situation. However, now, they have established their reputation, and people recognize the efficacy of PMC medicines. Lokesh (Keonjhar) and Suraj (Angul) revealed that despite physicians' distrust of PMC, people are gradually shifting to the PMC as they are finding it efficacious. This means that consumers are challenging the brand hegemony and physicians' control over their decisions regarding the right medicine for them. In the long run, PMBJP's objective of challenging the preference for branded generics is expected to significantly transform the Indian healthcare system.

Developing countries have adopted several measures to deal with high medicine prices, but these have not been as effective as India's PMBJP scheme. Many Middle Eastern countries, such as Egypt, Kuwait, Jordan, Lebanon, Qatar, Saudi Arabia, and the United Arab Emirates, adopted the External Reference Pricing (ERP) policy.^43^ Bangladesh implemented a National Drug Policy (NDP) to make the medicine affordable. Brazil implemented the annual price adjustment to make essential medicine accessible to the people.^44^ PMBJP is unique among all these policies because it addresses the root cause of high medicine prices without disturbing the pharmaceutical market. The scheme is determined to change the community's perception that high price equals quality. It is unique in that it engages stakeholders from the community by providing employment.

## 5 Limitations of the study

Trust and satisfaction of the PMC customers are evaluated indirectly by the narration of the pharmacists who were only interview participants. Trust and satisfaction are personal opinions. Pharmacists could have observed that, but trust and satisfaction from secondary sources can be somewhat biased or not justifiable. Including customers of PMCs as stakeholders would be the right approach to understanding the efficacy of the PMC medicine and its perceived benefits.

There are certain methodological limitations in the study. The process of participant recruitment is loosely defined in the study. However, the interviewers have taken care to represent the reality of stakeholders. The owner of a PMC may not be a serving pharmacist in his PMC. There are hired pharmacists or pharmacist assistants managing the sales at the store counter. Therefore, in a few cases, the employed pharmacist was not sure about the source of information needed to start the PMC, motivation, and supply chain. Defined categories of the owner not serving as a pharmacist, hired pharmacist, and the owner himself as a serving pharmacist can add different perspectives in a study. The employed pharmacists added some values in the interview that are unique to them, for example, their experience of working in branded pharmacies before and PMCs now. They were sometimes more critical of the PMBJP scheme. A few crucial data remain untapped without considering the emphasis on the role of employed pharmacists as potential participants while designing the interview guide.

There were some exceptionally performing PMCs, which were crowded by customers who refused to participate in the study as they did not have time to entertain the researchers. High-performing PMCs may have contributed vigorously to the present research as they were catering to the high demand for PMC medicines. Such PMC representatives should be accommodated by making necessary changes in the data collection plan. If these limitations are addressed, the new findings may reveal new dimensions in PMC research. The findings will highlight the specific issues in the area. However, as with other qualitative studies, the generalizability of the findings will remain limited.

## 6 Conclusion

The modification in the nature of PMC ownership post-2015 was undoubtedly a good decision to ensure the widespread expansion of PMCs in the Odisha and the rest of the country. The change in ownership for PMC expansion aligns with its objective to provide affordable medicine to all sections of the population. This transition has proven to be successful in making PMCs self-sustaining, but at the same time, the central government has failed to make a sustainable plan for the pre-2015 PMCs. The Red Cross-owned PMCs were facing a crisis situation in the province because of district collectors' intervention. Their number, too, was very low compared to private PMCs that started post-2015. Instead of leaving them to their fate, the central government should come forward with a plan to support or stop such PMCs in the province. The government can make them independent of district collectors' interventions by handing over the financial management to the NGO in the contract.

The ambiguity in the minds of consumers and physicians regarding the authenticity and efficacy of unbranded generic medications must be addressed to ensure that they get prescribed appropriately. Only price difference is not enough to differentiate PMC medicines from branded products. To promote unbranded molecule/salt-based generic medication, PMC has to show up as a recognizable name opposite to market generic brands. Advertisements are the most suitable means of achieving this purpose. ‘PMC week’ (1–7 March) is declared to be celebrated every year. This week is a good opportunity to bring the PMCs to media attention and bring them to public notice.

Research has shown that PMC medicines are safe and efficacious; if physicians are concerned about PMC efficacy, they should look for evidence and then decide on its reliability. Evidence-based research should be promoted and encouraged by reputed research organizations across the country. Before drugs' efficacy comes the socioeconomic condition of patients. Therefore, instead of making no effort to gather evidence and relying on brands, physicians should first gather evidence of the least-cost drugs available in the market. PMC medicines are such types of drugs with a reputation that it is introduced as a public welfare scheme. Physicians could proactively welcome it for a larger population benefit. Despite the low cost, PMC medicines are taking longer to be accepted by patients. They need a trusted referral to use PMC medicine because health is crucial, and no one wants to take risks. Physicians' referral is required to strengthen trust in PMC medicines.

Further, the issues of regional disparity in PMC provisions must be addressed in order to reach every citizen. The distribution of PMCs across the province is unequal, with some regions having far fewer PMCs than others. This raises concerns about the scheme's ability to serve its intended beneficiaries, particularly the poor. To stop the regional disparity, the government can introduce a cap system; for example, one PMC will be provided per 10,000 population.

Finally, patients' brand loyalty and trust in branded medicines pose a challenge to the adoption of PMC medicines. However, over time, people are gradually shifting towards PMCs as they recognize their efficacy.

In summary, while the PMBJP scheme has made significant progress, it faces various challenges related to ownership, distribution, and competition with branded medicines. These challenges require continuous evaluation and adaptation to ensure that the scheme effectively serves the population's healthcare needs, particularly the underserved and economically disadvantaged.

## CRediT authorship contribution statement

**Ranjit Kumar Dehury:** Writing – review & editing, Writing – original draft, Visualization, Validation, Supervision, Resources, Project administration, Methodology, Investigation, Funding acquisition, Formal analysis, Data curation, Conceptualization. **Imteyaz Ahmad:** Writing – review & editing, Writing – original draft, Visualization, Validation, Software, Project administration, Methodology, Investigation, Formal analysis, Data curation, Conceptualization. **Manas Ranjan Behera:** Writing – review & editing, Writing – original draft, Visualization, Project administration, Investigation, Conceptualization. **Varalakshmi Manchana:** Writing – review & editing, Writing – original draft, Visualization, Software, Formal analysis, Data curation, Conceptualization. **Parthsarathi Dehury:** Writing – review & editing, Writing – original draft, Visualization, Validation, Supervision, Software, Project administration, Formal analysis, Data curation, Conceptualization. **Deepanjali Behera:** Writing – review & editing, Writing – original draft, Visualization, Validation, Formal analysis, Data curation, Conceptualization. **Nafisa Vaz e Desouza:** Writing – review & editing, Writing – original draft, Visualization, Formal analysis, Data curation, Conceptualization.

## Declaration of competing interest

There is no conflict of Interest of any of the authour

## Appendix A Supplementary data

Interview guide to assess People's Medicine Centre. Image 1

Consolidated criteria for reporting qualitative studies (COREQ): 32-item checklist. Image 2
