# Supplementary material for: Genesis of People’s Medicine Centre (PMC) for popularisation of generic medicine: A critical qualitative inquiry
Source: Explor Res Clin Soc Pharm. 2024 May 22;14:100455. doi: 10.1016/j.rcsop.2024.100455 (PMC11167389; doi:10.1016/j.rcsop.2024.100455)
Supplement: Supplementary file 1 — Interview guide to assess People's Medicine Centre. [file mmc1.docx]

**Suplementary Materials**

**Interview guide for the People's Medicine Centre**

1. How you were informed about the People's Medicine Centre?
2. What is the ownership nature of this People's Medicine Centre? (Government, NGO, charitable, trust, private hospital, physician, pharmacist, or individual entrepreneur)
3. What motivated you to open a People's Medicine Centre?
4. Who most benefits from the People's Medicine Centre?
5. Is there any loss to anyone caused by the People's Medicine Centre?
6. What is the difference between generic and branded medicines?
7. How is the market competition in the People's Medicine Centre business? At what margin do you sell medicines?
8. How the PMC medicine price and private pharmacist/entrepreneurs share of profit is determined?
9. Do customers consult with you about medicine quality, prices, physician's prescription, self-medication etcetera? Is there a regular pharmacist (B. Pharma/ D. Pharma degree holder) in your store? If no, then how do you help them?
10. Do you think all generic treatment can be as effective as branded medicine treatment?
11. How is the general practitioners/ physician attitude towards PMC medicines?
12. How the PMBJP scheme can be more efficiently used while targeting patients/consumers?
13. How the PMBJP scheme can be more supportive to the store owners and its own sustenance in the future?
