# Supplementary material for: Genesis of People’s Medicine Centre (PMC) for popularisation of generic medicine: A critical qualitative inquiry
Source: Explor Res Clin Soc Pharm. 2024 May 22;14:100455. doi: 10.1016/j.rcsop.2024.100455 (PMC11167389; doi:10.1016/j.rcsop.2024.100455)
Supplement: Supplementary file 2 — Consolidated criteria for reporting qualitative studies (COREQ): 32-item checklist. [file mmc2.docx]

**Suplementary Materials**

Consolidated criteria for reporting qualitative studies (COREQ): 32-item checklist

| **No** | **Item** | **Guide questions/description** | **Remarks** |
| --- | --- | --- | --- |
| **Domain 1: Research team and reflexivity** | | | |
| Personal Characteristics | | | |
| 1 | Interviewer/facilitator | Which author/s conducted the interview or focus group? | Imteyaz Ahmad (2^nd^) & Parthsarathi Dehury (5^th^) |
| 2 | Credentials | What were the researcher's credentials? *E.g. PhD, MD* | M.Phil (2^nd^) & Ph.D. (5^th^) |
| 3 | Occupation | What was their occupation at the time of the study? | Research |
| 4 | Gender | Was the researcher male or female? | Male |
| 5 | Experience and training | What experience or training did the researcher have? | They have been part of various public health research projects in the last eight years. |
| Relationship with participants | | | |
| 6 | Relationship established | Was a relationship established prior to study commencement? | No |
| 7 | Participant knowledge of the interviewer | What did the participants know about the researcher? e*.g. personal goals, reasons for doing the research* | Researchers introduced themselves as researchers affiliated with the University of Hyderabad and carrying out a project on PMCs in Odisha. |
| 8 | Interviewer characteristics | What characteristics were reported about the interviewer/facilitator? e.g. *Bias, assumptions, reasons and interests in the research topic* | Primarily, interviewers were regarded as government agents. |
| **Domain 2: study design** | | | |
| Theoretical framework | | | |
| 9 | Methodological orientation and theory | What methodological orientation was stated to underpin the study? *e.g. grounded theory, discourse analysis, ethnography, phenomenology, content analysis* | Ethnography |
| Participant selection | | | |
| 10 | Sampling | How were participants selected? *e.g. purposive, convenience, consecutive, snowball* | Purposive and convenience |
| 11 | Method of approach | How were participants approached? e*.g. face-to-face, telephone, mail, email* | Face-to-face |
| 12 | Sample size | How many participants were in the study? | Seventeen |
| 13 | Non-participation | How many people refused to participate or dropped out? Reasons? | Six, due to busy hours, absence of PMC owner, and shutdown outlet. |
| Setting | | | |
| 14 | Setting of data collection | Where was the data collected? e*.g. home, clinic, workplace* | Workplace |
| 15 | Presence of non-participants | Was anyone else present besides the participants and researchers? | Yes |
| 16 | Description of sample | What are the important characteristics of the sample? *e.g. demographic data, date* | Pharmacy professionals, adult male and female, located in Odisha. |
| Data collection | | | |
| 17 | Interview guide | Were questions, prompts, guides provided by the authors? Was it pilot tested? | Yes, an interview guide was used. However, it was not pilot-tested but reviewed and revised by the principal investigator of the project. |
| 18 | Repeat interviews | Were repeat interviews carried out? If yes, how many? | No |
| 19 | Audio/visual recording | Did the research use audio or visual recording to collect the data? | Yes, interviews were audio recorded. |
| 20 | Field notes | Were field notes made during and/or after the interview or focus group? | No |
| 21 | Duration | What was the duration of the interviews or focus group? | Between 9 to 33 minutes |
| 22 | Data saturation | Was data saturation discussed? | Yes |
| 23 | Transcripts returned | Were transcripts returned to participants for comment and/or correction? | No |
| **Domain 3: analysis and findings** | | | |
| Data analysis | | | |
| 24 | Number of data coders | How many data coders coded the data? | One |
| 25 | Description of the coding tree | Did authors provide a description of the coding tree? | Yes |
| 26 | Derivation of themes | Were themes identified in advance or derived from the data? | Yes, themes were identified in advance. |
| 27 | Software | What software, if applicable, was used to manage the data? | The free version of InqScribe for transcribing and the trial version of MAXQDA for thematically arranging the data |
| 28 | Participant checking | Did participants provide feedback on the findings? | No |
| Reporting | | | |
| 29 | Quotations presented | Were participant quotations presented to illustrate the themes / findings? Was each quotation identified? e*.g. participant number* | Yes |
| 30 | Data and findings consistent | Was there consistency between the data presented and the findings? | Yes |
| 31 | Clarity of major themes | Were major themes clearly presented in the findings? | Yes |
| 32 | Clarity of minor themes | Is there a description of diverse cases or discussion of minor themes? | Yes |

Source: Tong et al. (2007) ()
